# Human herpesvirus reactivation and its potential role in the pathogenesis of post-acute sequelae of SARS-CoV-2 infection

**DOI:** 10.1007/s11357-024-01323-9

**Published:** 2024-08-29

**Authors:** Zsófia Gáspár, Bálint Gergely Szabó, Andrea Ceglédi, Botond Lakatos

**Affiliations:** 1 School of PhD Studies, Semmelweis University, Üllői Street 26, 1085 Budapest, Hungary; 2 South Pest Central Hospital, National Institute of Haematology and Infectious Diseases, Albert Flórián Street 5-7, 1097 Budapest, Hungary; 3 Departmental Group of Infectious Diseases, Department of Internal Medicine and Haematology, Semmelweis University, Albert Flórián Street 5-7, 1097 Budapest, Hungary

**Keywords:** COVID-19, SARS-CoV-2, PASC, POTS, Brain fog, EBV, CMV, Herpesvirus

## Abstract

The emergence of SARS-CoV-2 has precipitated a global pandemic with substantial long-term health implications, including the condition known as post-acute sequelae of SARS-CoV-2 infection (PASC), commonly referred to as Long COVID. PASC is marked by persistent symptoms such as fatigue, neurological issues, and autonomic dysfunction that persist for months beyond the acute phase of COVID-19. This review examines the potential role of herpesvirus reactivation, specifically Epstein-Barr virus (EBV) and cytomegalovirus (CMV), in the pathogenesis of PASC. Elevated antibody titers and specific T cell responses suggest recent herpesvirus reactivation in some PASC patients, although viremia is not consistently detected. SARS-CoV-2 exhibits endothelial trophism, directly affecting the vascular endothelium and contributing to microvascular pathologies. These pathologies are significant in PASC, where microvascular dysfunction may underlie various chronic symptoms. Similarly, herpesviruses like CMV also exhibit endothelial trophism, which may exacerbate endothelial damage when reactivated. Evidence suggests that EBV and CMV reactivation could indirectly contribute to the immune dysregulation, immunosenescence, and autoimmune responses observed in PASC. Additionally, EBV may play a role in the genesis of neurological symptoms through creating mitochondrial dysfunction, though direct confirmation remains elusive. The reviewed evidence suggests that while herpesviruses may not play a direct role in the pathogenesis of PASC, their potential indirect effects, especially in the context of endothelial involvement, warrant further investigation.

## Introduction

The emergence of SARS-CoV-2 (severe acute respiratory syndrome coronavirus 2) in 2019 has led to a global pandemic with profound health, social, and economic impacts [1]. As of mid-2024, COVID-19 has resulted in over 775 million confirmed cases and more than 7 million deaths worldwide [2]. The virus has significantly impacted global mortality rates, particularly among older adults and those with underlying health conditions[3–6]. The mortality rate for COVID-19 varies widely depending on age, with older adults being disproportionately affected [3]. For instance, individuals aged 65 and older account for over 80% of COVID-19 deaths in many countries [7]. Older adults are particularly vulnerable to severe outcomes from COVID-19 due to several factors, including the prevalence of comorbidities such as cardiovascular disease, diabetes, and chronic respiratory conditions, which exacerbate the severity of the infection [3–6, 8]. Additionally, the efficiency of the immune system declines with age, a phenomenon known as immunosenescence, making it harder for older adults to mount an effective immune response to the virus [3, 9–11].

While the acute phase of COVID-19 has been extensively studied, increasing attention is being given to a significant subset of individuals who experience persistent symptoms and health issues long after the initial infection, known as post-acute sequelae of SARS-CoV-2 infection (PASC), or Long COVID [12–19]. The World Health Organization (WHO) characterizes PASC as the persistence or emergence of new symptoms three months after the initial SARS-CoV-2 infection, with these symptoms lasting for at least two months without any other identifiable cause [12]. However, various studies use differing definitions and associate this condition with a wide range of symptoms, durations, and risk factors, complicating comparisons and summarization of evidence (Table 1) [20].

**Table 1:** Definitions of PASC and associated symptoms according to the cited studies

Reference	No. of patients with PASC	Inclusion criteria	PASC symptoms
[21]	24,434	Included patients according to the NICE definition of PASC and a PASC score ≥ 12	Fatigue, musculoskeletal pain, headache, cough, loss of smell or taste, fever, post-exertional malaise, brain fog, anxiety, and chest pain
[76]	84	Patients with a SARS-CoV-2 infection confirmed by a polymerase chain reaction and fulfilling the WHO criteria for PASC	Cognitive deficits, fatigue, headache, myalgia, pain syndromes, sleep disorder, sensory dysfunction, vertigo, loss of smell/taste
[38]	133,366	Patients who tested positive for COVID-19 at least 21 days before enrolment and reported any long COVID symptoms were included	Fatigue, myalgia, dementia, encephalopathy, amnesia
[75]	9	SARS-CoV-2-infected and deceased patients with a SARS-CoV-2 infection confirmation by a nasal swab polymerase chain reaction at the time of death	Neurocognitive impairment
[37]	65	Referred to the WHO criteria for PASC	Fatigue, muscle/joint pain, dyspnea, cough, chest pain, palpitations, sleep disturbance, loss of smell/taste, headache, brain fog, memory problem
[79]	357	Patients who tested positive for COVID-19 at least 21 days before enrolment and reported any long COVID symptoms were included	Fatigue, insomnia, headaches, myalgia, confusion/brain fog, weakness, rash, pharyngitis, abdominal pain, tinnitus, fever over 101° F, neck lymphadenopathy, or mild-to-moderate hearing loss
[199]	400	Referred to the WHO criteria for PASC	Fatigue, weakness, shortness of breath, altered sense of taste/smell, nasal congestion, cough, chest pain, body ache, headache, brain fog, elevated blood pressure, sleep disorders
[80	101	Patients with a confirmed or probable SARS-CoV-2 infection and who have persistent symptoms lasting more than six weeks following the initial infection	Fatigue, post-exertional malaise, and a variety of cognitive and autonomic dysfunctions
[182]	278	Main inclusion criteria were a polymerase chain reaction confirmed SARS-CoV-2 infection and at least 6 months between the infection and the inclusion	Neurological disturbances, extreme fatigue, post-exertional malaise, postural orthostatic tachycardia syndrome
[40]	280	Any adult with a confirmed history of SARS-CoV-2 infection, as identified by nucleic acid amplification testing, was eligible to enroll in the study more than 14 days after symptom onset, regardless of whether they experienced acute or post-acute symptoms, and was subsequently followed at approximately 4-month intervals	Referred to CDC COVID-19 symptom and Patient Health Questionnaire somatic symptom lists
[83]	30	Referred to the NICE criteria for PASC	Fatigue, post‐exertional malaise, autonomic dysfunction, orthostatic intolerance
[39]	121	Patients with a confirmed SARS-CoV-2 infection and who have persistent symptoms lasting more than six months following the initial infection	Pain, dyspnea, fatigue, reduced daily activities, discommunication
[28]	14	Patients who experienced a clinically suspected or confirmed SARS-CoV-2 infection and have persistent symptoms	Respiratory symptoms, neurological symptoms, fatigue, pain, mental dysfunction, cardiovascular dysfunction, post-exertion symptoms, cognitive dysfunction, orthostatic intolerance
[82]	90	Referred to the WHO criteria for PASC	Fatigue, memory/concentration issues, muscle aches, loss of smell/taste, emotional distress, cognitive impairment
[195]	14	Patients who had a confirmed SARS-CoV-2 infection and continue to experience persistent neurological symptoms	Fatigue, respiratory issues, myalgia, neurological manifestations (brain fog)

Patients with prolonged symptoms after documented COVID-19 can be categorized into three subgroups [1, 20, 21]. The first subgroup includes individuals hospitalized for severe SARS-CoV-2 infection who display signs of post-intensive care syndrome [20, 22]. The second subgroup consists of individuals with previously undiagnosed chronic comorbidities, whose symptoms may have emerged as an indirect consequence of the pandemic’s health, social, and economic impacts (e.g., isolation, lifestyle changes) [1, 20, 21]. The third subgroup involves patients experiencing prolonged symptoms after the acute phase of SARS-CoV-2 infection, a condition defined in the literature as PASC [20].

The National Institute for Health and Care Excellence (NICE) defines PASC as either ongoing symptomatic COVID-19 in individuals who continue to experience symptoms between 4 and 12 weeks after the onset of acute symptoms or as post-COVID-19 syndrome in individuals who continue to experience symptoms for more than 12 weeks after the onset of acute symptoms [23]. Alternatively, the WHO defines PASC as a condition in individuals with suspected or confirmed SARS-CoV-2 infection who exhibit persistent symptoms lasting for at least two months without an alternative pathophysiological explanation [12, 21]. In the literature, the terms Long COVID, long-hauler, post-COVID condition, and PASC frequently overlap [12]. Additionally, due to similar prolonged symptomatology and the post-viral syndrome theory, myalgic encephalopathy/chronic fatigue syndrome (ME/CFS) has also been compared with PASC [20].

### Symptoms of PASC

Patients report various symptoms as part of PASC, including neurological symptoms (commonly referred to as brain fog), fatigue, sleep disturbances, memory disturbances, headaches, autonomic dysfunction such as postural orthostatic tachycardia syndrome (POTS), musculoskeletal pain, post-exertional malaise, chest pain, cough, loss of smell or taste, and anxiety [20, 21, 24]. While many of these symptoms are difficult to define or directly link to a recent SARS-CoV-2 infection, dysautonomia and neurological symptoms have measurable alterations [20]. One of the most commonly documented symptoms of PASC is “brain fog,” which typically refers to a lack of focus, impaired short-term memory, and diminished cognitive sharpness in affected patients [25]. Among the autonomic dysfunction symptoms of PASC, POTS is characterized by a tachycardic state experienced by patients upon standing up from a lying position [26, 27]. The pathophysiology of POTS is diverse, involving factors such as excessive sympathetic activity, impaired peripheral autonomic function, volume dysregulation, and cardiovascular or autoimmune dysfunction [20]. Recently, POTS has been linked to PASC [20, 26, 27]. The typical manifestation of POTS includes palpitations (heart rate increase of > 30 beats per minute without a blood pressure drop), dizziness, and shortness of breath, typically occurring after standing up or walking [20]. Campen et al. described that in patients infected with SARS-CoV-2, high sympathetic activity is present during the earlier stages of the clinical course, manifesting as POTS [28]. Alterations of sympathetic activity persist while further PASC symptoms develop and gradually decrease over time [28].

### Prevalence of PASC

Evaluating the prevalence of PASC is challenging due to heterogeneous definitions, symptomatology, and the lack of consensus [21]. Conservative estimates based on WHO data suggest that approximately 10–20% of individuals infected with SARS-CoV-2 subsequently develop this condition [12]. However, the prevalence may be significantly higher in certain populations, with studies indicating that close to two-thirds of COVID-19 survivors may develop PASC. This higher prevalence is particularly observed among patients who experienced severe acute illness, were hospitalized, or had pre-existing health conditions [4, 13, 15, 16, 29–36]. Age is a significant risk factor for the development of PASC [15, 37]. Previous studies indicate that over one-third of patients aged 65 and older who have had COVID-19 develop persistent symptoms lasting more than 6 months, leading to a considerable reduction in quality of life [38, 39]. However, these symptoms are often attributed to pre-existing chronic comorbidities by healthcare professionals, complicating the direct attribution to PASC [37]. Furthermore, elderly patients might be less likely to seek professional care compared with younger patients with persistent or new-onset post-acute infection symptoms, contributing to a lower rate of PASC diagnosis in this population [37].

### Potential mechanisms involved in the pathogenesis of PASC

The potential causes and pathophysiological mechanisms of PASC remain unknown, though several theories exist, including microvascular dysfunction, persisting low-grade neuroinflammation, mitochondrial damage and dysfunction, autoimmune processes, SARS-CoV-2 viral persistence, and immune activation, either individually or in combination [40, 41].

#### Role of microvascular endothelial dysfunction

Microvascular dysfunction has emerged as a significant factor in the pathogenesis of neurological sequelae of COVID-19 and PASC. The endothelial trophism of SARS-CoV-2 directly impacts the microvasculature, leading to widespread endothelial damage and inflammation [42–44]. This damage is often characterized by endothelial cell activation, microthrombosis, and increased vascular permeability, contributing to a range of long-term symptoms [44–51]. In patients with COVID-19, persistent endothelial dysfunction has been observed long after the acute phase of the infection [47–50, 52–63]. This dysfunction is associated with impaired neurovascular coupling responses, which are critically relevant to cognitive impairment [55]. Neurovascular coupling, the mechanism by which neural activity is matched with blood flow ensures that active regions of the brain receive adequate oxygen and nutrients [64]. In PASC patients, the disruption of this process due to endothelial dysfunction can lead to inadequate cerebral perfusion. This mismatch between neuronal demand and blood supply is believed to contribute directly to symptoms such as brain fog, memory disturbances, and reduced cognitive function often reported in PASC patients. Additionally, microvascular injury may lead to the disruption of the blood–brain barrier (BBB), allowing inflammatory mediators to enter the central nervous system and contribute to neuroinflammation [46].

#### Role of neuroinflammation

Emerging evidence suggests that persisting low-grade neuroinflammation plays a critical role in the pathogenesis of PASC [45, 65]. Studies have shown that individuals with PASC often exhibit markers of ongoing inflammation, which may underlie many of the neurological and cognitive symptoms associated with this condition [42, 66–69]. For instance, research has identified elevated levels of pro-inflammatory cytokines and chemokines, such as IL-6, TNF-alpha, and CCL11, in the cerebrospinal fluid and blood of PASC patients, indicating a persistent inflammatory state in the central nervous system [70–75]. Persisting microglial activation, a hallmark of neuroinflammation, has been observed in the brain tissue samples following mild SARS-CoV-2 infection [75]. Microglia activation was significantly higher among human patients with PASC and neurological symptoms, compared with those with PASC without neurological symptoms [75].This chronic microglia activation can lead to synaptic dysfunction, neuronal injury, and impaired neurogenesis, contributing to the symptoms of brain fog, memory disturbances, and cognitive decline reported by many PASC patients. Besides microglia reactivity, impaired hippocampal neurogenesis, myelin loss, and a reduced number of oligodendrocytes were identified in brain tissues derived from PASC patients [75]. Moreover, the breakdown of the BBB has been implicated in PASC [46]. The BBB disruption allows peripheral inflammatory mediators to enter the brain, exacerbating local neuroinflammatory processes. This mechanism further supports the hypothesis that persistent low-grade neuroinflammation is a central feature of PASC pathogenesis. A recent study examined cerebrospinal fluid (CSF) samples from PASC patients with neurological symptoms [76]. Elevated protein levels were found in a quarter of the patients, and 13% exhibited blood–brain barrier dysfunction measured via the CSF/serum albumin quotient [76]. Overall, these findings underscore the importance of neuroinflammation in PASC and suggest potential therapeutic targets for managing PASC symptoms, including anti-inflammatory treatments and strategies to restore BBB integrity [77, 78].

#### Connection between microvascular endothelial dysfunction, neuroinflammation, and herpesvirus reactivation

A potential contributor to persistent neuroinflammation in PASC is the reactivation of latent herpesviruses, such as Epstein-Barr virus (EBV) and cytomegalovirus (CMV). Both EBV and CMV have known neurotropic and endothelial-tropic properties, allowing them to infect and persist in the central nervous system and the vascular endothelium. Reactivation of these viruses under conditions of immune stress, such as during or after an acute SARS-CoV-2 infection, can exacerbate the inflammatory environment within the brain. The presence of these viruses can trigger additional immune responses, further activating microglia and perpetuating the cycle of neuroinflammation [79–83]. Emerging evidence underscores the interconnected roles of herpesvirus infection/reactivation and microvascular endothelial dysfunction as well [84–91]. Reactivated EBV and CMV can infect endothelial cells and promote a pro-inflammatory state, thereby perpetuating the cycle of endothelial dysfunction and neuroinflammation. This interplay between viral reactivation and endothelial damage may contribute to the persistence and severity of PASC symptoms.

This review aims to provide a comprehensive summary of the potential role of various herpesvirus infections in the pathogenesis of PASC, highlighting the complex interconnections between microvascular endothelial dysfunction, neuroinflammation, and viral reactivation ^40^. Understanding these mechanisms is crucial for developing targeted therapeutic strategies to mitigate the long-term impacts of PASC.

## Background on herpesviruses and their reactivation

*Herpesviridae* is a large family of double-stranded DNA viruses that affect humans and animals [92]. Key members affecting only humans are divided into three subfamilies: alpha (herpes simplex virus type 1 and 2, and varicella-zoster virus), beta (CMV, human herpesvirus 6 [HHV-6], and human herpesvirus 7), and gamma (EBV and Kaposi’s sarcoma-associated herpesvirus/human herpesvirus 8) [92]. Transmission occurs via direct contact, respiratory routes, or body fluids, with viral replication and assembly taking place within the host cell [92].

### Clinical phases of herpesvirus infection

Human herpesvirus infection is characterized by three distinct clinical phases: the acute phase, latent infection, and reactivation [93]. The acute phase involves continuous viral replication, viral assembly, and a cytolytic mechanism, during which the virus is released from the cell. This phase primarily involves epithelial cells and is controlled by the adaptive immune system [93]. Subsequently, the virus enters a latent phase in specific cell types, with the viral genome present in the host cell nucleus without significant active replication [93].

#### Biological mechanisms of latency

The latency phase of herpesvirus infection is a complex process that enables the virus to persist in the host for extended periods without causing active disease. This phase is marked by the maintenance of the viral genome in a dormant state within host cells, where it evades the host immune system. Several biological mechanisms contribute to the establishment and maintenance of viral latency. Latency-associated transcripts (LATs) are non-coding RNAs expressed during the latent phase of herpesvirus infection [94, 95]. These transcripts play a crucial role in maintaining latency by inhibiting apoptosis and modulating the host cell’s stress response, thereby ensuring the survival of infected cells and the viral genome [94]. The viral genome undergoes epigenetic modifications such as methylation and histone modification, which silence the expression of lytic genes and maintain the virus in a quiescent state [96–99]. These epigenetic changes prevent the reactivation of the virus under normal conditions but allow for quick reactivation when triggered by specific stimuli [96–99]. During latency, herpesviruses employ multiple strategies to evade the host immune system [100–103]. For example, they downregulate the expression of major histocompatibility complex (MHC) molecules on the surface of infected cells, reducing their recognition and destruction by cytotoxic T lymphocytes [104]. Additionally, latent viruses produce proteins that inhibit the presentation of viral antigens and the activation of immune responses [104–107].

The mechanisms of viral latency also involve microRNA (miRNA) regulation [105, 108–111]. Herpesviruses encode miRNAs that can modulate both viral and host gene expression. These miRNAs can downregulate the expression of viral lytic genes and host immune response genes, thereby maintaining latency and preventing the activation of the immune system against the virus [105, 108–111]. Herpesviruses establish latency in specific cellular reservoirs that are less likely to be targeted by the immune system [112–115]. CMV primarily establishes latency in myeloid lineage cells, including monocytes, macrophages, and their CD34 + progenitor cells in the bone marrow. During latent infection, the viral genome persists in these cells without producing infectious virions. CMV can reactivate in response to immunosuppression or cellular differentiation, leading to viral replication and dissemination. EBV primarily targets B lymphocytes for latency, especially memory B cells. HHV-6 establishes latency in a variety of cell types, including monocytes, macrophages, and CD4 + T lymphocytes. These cellular reservoirs provide a protected environment for the viruses, allowing them to evade immune surveillance and persist for the lifetime of the host. During latent infection, some fraction of latently infected cells may still produce low levels of virions, which can activate a herpesvirus-specific T-cell response [93]. These herpesvirus-specific T cells can induce inflammatory responses at mucosal surfaces and become transiently activated during secondary infections, potentially modulating immune responses to other antigens [93]. Understanding the intricate mechanisms of herpesvirus latency is crucial for developing strategies to manage and treat herpesvirus-associated diseases, particularly in the context of co-infections such as SARS-CoV-2.

### Epstein-Barr virus (EBV)

EBV is an oncogenic virus transmitted through close contact, replicating within the oropharyngeal epithelium and B cells. In immunocompetent patients, primary infection manifests either asymptomatically or as the classic mononucleosis syndrome [92]. The innate immune system recognizes EBV antigens through various pattern recognition receptors, subsequently activating the adaptive immune system and eliciting a virus-specific immune response. Activated B cells and CD8 + T cells recognize lytic viral antigens and latent cells [116]. After primary infection, the virus becomes latent in memory B cells [116]. During latency, EBV infected cells adopt one of several latency programs, restricting viral genome expression and maintaining non-replicating regions in a highly methylated state [116]. Virus-associated latent membrane protein 1 (LMP1) and latent membrane protein 2A (LMP2A) downregulate antigen processing [116, 117]. EBV-encoded microRNAs create an immunosuppressive environment, and LMP proteins contribute to prolonged B cell survival, continuous activation, and apoptosis avoidance through signaling pathways [116].

Reactivation of latent EBV can occur under immunocompromised conditions, acute stressors, or concurrent acute infections [40]. In most latent cells, the EBV genome is present without significant replication; however, some viral genes continue to be expressed, driving oncogenesis [118]. Proteins responsible for the cytolytic phase also play a role in driver mutations [118]. EBV is associated with various lymphoproliferative diseases (e.g., Hodgkin lymphoma, some non-Hodgkin lymphomas, Burkitt lymphoma) and epithelial cell malignancies (e.g., nasopharyngeal carcinoma) [118]. Also, in HIV-associated lymphomas, especially primary central nervous system lymphomas EBV in malignant cells can be detected in approximately 40–100% of cases[119]. The exact pathogenesis of primary central nervous system lymphomas without systemic involvement remains not fully understood[120]. However, potential mechanisms have been suggested, including the production of adhesion molecules like BCA-1 by malignant B cells, facilitating their migration to the central nervous system[120]. Additionally, STAT-6 and interleukin-4 have been implicated in tumor progression[120].

Additionally, EBV is linked to autoimmune diseases, including systemic lupus erythematosus (SLE), rheumatoid arthritis, Sjögren’s syndrome, and multiple sclerosis [117, 121]. The mechanisms and evidence behind the association between EBV and autoimmune diseases involve several interrelated processes. First, EBV-associated antibodies can cross-react with SLE-specific autoantigens, a phenomenon known as molecular mimicry, which allows autoantibodies to target and damage human tissues [117]. Furthermore, EBV latency proteins can influence B cell survival, immunoglobulin production, and cytokine production, leading to a dysregulated immune system and triggering autoimmune pathways [117]. Studies have also indicated a reduced EBV-specific CD8 + T-cell response coupled with elevated CD4 + T-cell levels, indicating a poor EBV-specific immune response^117^. Additionally, patients with SLE often exhibit elevated levels of viral nucleic acids or EBV antigen titers, providing further evidence of the association [117]. Similar associations can be observed between multiple sclerosis (MS) and EBV infection [121]. Individuals infected with EBV have a 30-fold increased risk of developing MS compared with those who are EBV-negative [121]. A high EBV antibody titer following infection is a strong predictor for the development of MS [121]. Pathogenesis theories of MS highlight a lag period after primary EBV infection during which clonal B cell lineages and a poor CD8 + T-cell response develop [121]. This period also sees the strengthening of molecular mimicry and epitope spreading, contributing to the autoimmune processes involved in MS development[121].

### Cytomegalovirus (CMV)

CMV is a beta-herpesvirus that typically manifests asymptomatically or as mononucleosis syndrome [92]. Primary infection is usually self-limiting and requires only supportive care [122]. CMV seroprevalence increases with age, approaching nearly 100% in developing countries [122]. During primary infection, a robust immune response is generated, involving both the innate and adaptive immune systems [123]. The adaptive immune response includes the production of specific neutralizing antibodies and CD4 + and CD8 + T cells [124]. Despite the strong primary immune response, CMV establishes latency through immunomodulation, which can involve modulation of direct NK cell recognition or interferon responses. CMV also produces an interleukin-10 (IL-10) homologue, which inhibits Th1-mediated monocyte activation and major histocompatibility complex II (MHC-II) presentation [124]. CMV genes expressed during the lytic phase interfere with MHC-I and MHC-II, inhibiting adaptive immune system activation [124].

After primary infection, CMV latency is predominantly established in myeloid cells and their CD34 + bone marrow progenitors, as well as in epithelial and mesenchymal cells [122, 124]. During latency, no active virion production occurs; only latency-associated genes and proteins (LUNA, UL138, US28, LAvIL-10) are expressed, and the primary promoter responsible for the lytic cycle is silenced [123]. The proteins produced by latency-associated genes elicit CD4 + T cell responses [124]. CD4 + T cells can recognize latently infected monocytes and restrict MHC-II-associated cytotoxicity [124]. Similar to EBV infection, where LMP1 initiates the IL-10 pathway, CMV latency-associated CD4 + T cells also produce IL-10 and TGF-ß, downregulating immune activation [123, 124]. From latency, CMV reactivation can occur, either iatrogenically or due to medical conditions, leading to disseminated disease with multi-organ involvement [92].

Primary CMV infection can also occur congenitally. During pregnancy, maternal infection may result from the reactivation of a latent virus or from reinfection through close contact with a susceptible individual[125]. Maternal infection leads to viremia, allowing the virus to spread transplacentally to the foetus[126]. Congenital CMV is the leading cause of congenital infections, in a long-term contributing to visual or sensorineural hearing impairment, intellectual disability, and cerebral palsy[125, 127, 128]. Studies on congenital CMV have demonstrated that almost all cell types in the central nervous system are susceptible to CMV infection, with astrocytes and the microvasculature system particularly supporting the entire replication process[126].

### Human herpesvirus 6 (HHV-6)

Human herpesvirus 6 encompasses two distinct viruses: HHV-6A and HHV-6B[129]. Similar to other herpesvirus infections, HHV-6 targets and replicates within a wide range of cells primarily targeting CD4 + T-cells[130]. Primary infection, which is generally a mild, self-limiting illness, occurs almost exclusively due to HHV-6B during the first 3 years of life [130, 131]. Following primary infection, the virus establishes latency in the monocyte-macrophage system and T-cells [130].

Reactivation of HHV-6 can occur under various immunosuppressive conditions, most commonly associated with solid-organ or hematopoietic cell transplantation, leading to end-organ diseases such as myelosuppression, encephalitis, pneumonitis, and hepatitis [130, 132]. HHV-6 is also linked to a range of neurological conditions, including febrile seizures, mesial temporal lobe epilepsy, and encephalitis, attributed to its neurotropic properties [133, 134]. The virus exhibits neuroinvasive characteristics, activating oligodendrocytes and astrocytes, thereby creating a Th1-mediated proinflammatory state [134]. Additionally, HHV-6 binds to the CD46 receptor, contributing to an enhanced complement activation, decreased interleukin-10 production and increased interleukin-17 level, thereby promoting neuroinflammation and the development of neurological conditions [134]. HHV-6 has also been linked to myalgic encephalomyelitis/chronic fatigue syndrome (ME/CFS), although evidence remains inconclusive [81, 135].

### Herpesviruses and endothelial trophism

Herpesviruses, including CMV, EBV, and HHV-6, exhibit endothelial trophism, meaning they have a propensity to infect and persist in endothelial cells [85, 87, 89, 90, 114, 136–150]. This endothelial infection plays a significant role in the pathogenesis and clinical manifestations of herpesvirus infections.

CMV is well-documented for its endothelial trophism [85, 90, 114, 136, 139–142, 149]. The virus can infect and establish latency in endothelial cells, leading to various vascular pathologies [85, 90, 114, 136, 139–142]. CMV infection of endothelial cells induces a pro-inflammatory state characterized by the expression of adhesion molecules and the secretion of cytokines and chemokines, which can promote leukocyte adhesion and transmigration [90]. This inflammation contributes to the development of atherosclerosis, transplant vasculopathy, and other vascular diseases [84, 89, 90, 151]. CMV infection was shown to cause endothelial dysfunction and potentially impair endothelial barrier function [152–154]. CMV infection likely also promotes the formation of microthrombi [155–157]. EBV also has the capability to infect endothelial cells [145–147]. In endothelial cells, EBV can induce changes that promote endothelial dysfunction and inflammation and contribute to the pathogenesis of various vascular diseases [145–147, 158]. HHV-6 is also known to infect endothelial cells [149]. HHV-6 infection in endothelial cells can lead to the production of pro-inflammatory cytokines and the upregulation of adhesion molecules, promoting an inflammatory response and leukocyte adhesion [150]. The endothelial trophism of these herpesviruses means that they can directly contribute to vascular inflammation and damage, which are central features in many of their associated diseases. Reactivation of herpesviruses in endothelial cells is thought to contribute to the exacerbation of a range of pathologies, particularly in immunocompromised individuals [138, 159–161].

### Risk factors for herpesvirus reactivation

Various risk factors can contribute to enhanced viral replication and gene expression, leading to the reactivation phase of herpesviruses [93]. These risk factors differ among *Herpesviridae* subfamilies but typically include oncohematological malignancies, steroid or other immunosuppressive treatments, chemotherapy, irradiation, local injury, other infections, UV light exposure, and hormonal imbalances [162]. Additionally, age-associated degradation of the immune system, particularly affecting cellular immunity, plays a significant role [163]. Immunosenescence refers to the functional decline of the immune system associated with aging, characterized by reduced responses to antigen stimuli and a decreased number of effective immune cells, increasing susceptibility to infections and auto-reactive pathways [163]. Age-related chronic low-grade inflammation creates a pro-inflammatory environment in various tissues, facilitating herpesvirus reactivation and contributing to low-level chronic inflammation, further exacerbating age-related immune mechanisms [164].

## Reactivation of herpesviruses in PASC

The reactivation of latent herpesviruses, such as EBV, CMV, and HHV-6, is a significant concern in patients with COVID-19 [82, 165–178]. A recent study conducted a comprehensive analysis of CMV seropositivity rates among patients with varying severities of SARS-CoV-2 infection [179]. This study revealed a notable trend: severely ill patients exhibited higher CMV seroprevalence compared to the general population [179], highlighting the intricate relationship between COVID-19 severity and the likelihood of herpesvirus reactivation. Previous studies identified that SARS-CoV-2 specific T cell receptors (TCRs) exhibited a robust response to CMV, indicating an immunomodulatory role of CMV in the pathogenesis of COVID-19 [180]. This was further substantiated by Frozza et al., who investigated CMV serology, CMV-specific T cells, and cytokine profiles in COVID-19 patients [181]. Their study demonstrated a Th17-dominated immune shift due to CMV infection, marked by elevated levels of CMV-specific CD4 + and CD8 + T cell responses and increased production of cytokines such as IFNγ, IL-17, and TNFα in both mild and severe COVID-19 cases [181]. Recent studies confirm associations between various herpesvirus infections and PASC [82, 182]. For instance, a notable study categorized previously SARS-CoV-2-infected patients into subgroups based on the severity of their post-COVID-19 conditions—mild, severe, or without chronic symptoms [182]. These groups were compared with healthy donors and patients with ME/CFS [182]. The study found a significantly higher IgG response against EBV and HSV-1 in the post-COVID-19 groups compared with healthy donors [182]. Additionally, elevated HHV-6 antibody titers were observed in the ME/CFS subgroup, suggesting a possible link between these viral infections and the persistence of symptoms in PASC patients [182].

Several factors associated with COVID-19, including immune dysregulation, systemic inflammation, and direct viral interactions, can trigger the reactivation of these latent viruses. COVID-19 is characterized by profound immune dysregulation [41], which can diminish the host’s ability to keep latent herpesviruses in check. This weakened immune surveillance can lead to the reactivation of these viruses. Studies have shown that severe SARS-CoV-2 infection can significantly suppress T-cell function[41], which is critical for controlling latent herpesvirus infections. Increased production of inflammatory cytokines induced by SARS-CoV-2 infection can also contribute to herpesvirus reactivation. Elevated levels of pro-inflammatory cytokines such as IL-6, TNF-alpha, and IFN-gamma can create an environment conducive to viral reactivation. These cytokines can reactivate herpesviruses by modulating the expression of viral genes and promoting the transition from latency to the lytic cycle. SARS-CoV-2 may also directly influence the reactivation of latent herpesviruses through molecular interactions. Additionally, the viral proteins of SARS-CoV-2 can interact with cellular pathways that regulate viral latency, thereby facilitating the reactivation of latent herpesviruses.

Reactivation of herpesviruses in the context of COVID-19 has significant clinical implications. Patients experiencing reactivation of EBV, CMV, or HHV-6 can present with a range of symptoms that overlap with or exacerbate those of PASC, such as fatigue, cognitive impairment, and inflammatory conditions.

### Role of CMV reactivation in the pathogenesis of PASC

CMV has been proposed as an indirect contributor to the long-term symptoms of PASC by inducing immune alterations, thereby aggravating immune dysregulation [183]. Primary CMV infection elicits a robust and highly differentiated CMV-specific T cell response, which persists as a significant proportion of the T cell repertoire, resulting in prolonged immune alteration [179, 180]. These specific T cells often exhibit signs of immunosenescence, such as the loss of CD28 expression and the accumulation of CD57 and KLRG1 [183]. This immune alteration may contribute to SARS-CoV-2 infection-induced dysregulation, facilitating CMV replication, while CMV-associated T cell changes reduce the efficacy of the immune response against SARS-CoV-2. Furthermore, through molecular mimicry, CMV can trigger autoimmune responses, inflammation, and tissue damage, exacerbating PASC symptoms [183]. CMV reactivation may play a role in neurocognitive dysfunction, brain fog, and musculoskeletal syndromes associated with PASC [183]. Although clinical trials have not demonstrated significant CMV viremia among PASC patients, elevated antibody titers suggesting viral reactivation are often present [40, 123].

CMV exhibits significant endothelial trophism. The virus can infect and establish latency in endothelial cells, leading to chronic endothelial dysfunction. Given the endothelial trophism of both SARS-CoV-2 and CMV, a plausible hypothesis is that the reactivation of CMV and/or other herpesviruses within endothelial cells may contribute to the pathogenesis of PASC. This hypothesis is supported by several observations. First, the presence of CMV in endothelial cells has been causally linked to the impairments of endothelium-dependent regulation of blood flow and endothelial barrier function [85, 90, 114, 136, 139–142]. This is particularly relevant given the emerging importance of microvascular impairments in the pathogenesis of PASC [45, 46, 50, 52, 61, 63, 65, 184–186]. Reactivation of CMV in endothelial cells may potentially cause a range of microvascular pathologies, which are significant factors in the development of PASC. Continuous viral presence and activity within endothelial cells can sustain a pro-inflammatory and pro-thrombotic state, contributing to ongoing symptoms such as fatigue, brain fog, and cardiovascular issues. CMV reactivation in endothelial cells can lead to the production of pro-inflammatory cytokines and chemokines, which enhance leukocyte adhesion and transmigration across the endothelial barrier. This inflammatory cascade could result in neurovascular damage, contributing to persistent low-grade neuroinflammation and impaired regulation of cerebral blood flow, conditions commonly observed in PASC patients. Moreover, CMV reactivation in the endothelial cells of the brain vasculature can compromise the integrity of the BBB. This compromised BBB allows peripheral inflammatory mediators and immune cells to infiltrate the central nervous system, exacerbating neuroinflammation. This exacerbated neuroinflammation is likely to contribute to neurocognitive symptoms such as brain fog and cognitive dysfunction. Importantly, the endothelial involvement in herpesvirus reactivation is expected to mirror the pathophysiological mechanisms seen in chronic conditions like ME/CFS, which is also associated with viral infections and endothelial dysfunction [26, 61, 138, 187–194]. The potential role of CMV reactivation in promoting these cerebromicrovascular and neurological abnormalities underscores the need for further studies to elucidate the exact mechanisms involved and to explore targeted therapeutic interventions. Understanding the consequences of CMV’s endothelial trophism and its implications for CMV reactivation is crucial for developing effective treatments for PASC. Further research is necessary to investigate the extent of CMV reactivation in endothelial cells and its direct contribution to the pathophysiology of PASC.

### Role of EBV reactivation in the pathogenesis of PASC

EBV reactivation has been implicated as a potential contributor to the pathogenesis of PASC [28, 40, 76, 79–81, 168, 172, 173, 178, 195–198]. EBV viremia can occur among hospitalized, critically ill COVID-19 patients and may predict the development of PASC [80]. Acute SARS-CoV-2 and EBV infections may interact under these circumstances [81]. The SARS-CoV-2 receptor, angiotensin-converting enzyme 2 (ACE2), promotes the Z transcriptional activator, enhancing latent EBV reactivation [81]. EBV reactivation, in turn, increases ACE2 expression on epithelial cells, promoting SARS-CoV-2 viral entry [81].

The reactivation of EBV in the context of COVID-19 and PASC has significant clinical implications [198]. Recent studies have explored the link between EBV reactivation and PASC [40]. One study conducted serologic testing for recent EBV and CMV infections on 280 adults with post-COVID-19 conditions [40]. Using logistic regression, the study found that fatigue and neurocognitive dysfunction were significantly associated with EBV early antigen-diffuse IgG (EA-D) positivity or high nuclear antigen (EBNA) levels [40]. In another study, the prevalence of EBV reactivation was investigated in patients 21–90 days and over 90 days post-SARS-CoV-2 infection [118]. Results indicated that 30.3% of these patients had elevated anti-EBV antibody titers, suggesting a similarity in symptomatology between EBV reactivation and post-COVID-19 conditions [118]. In contrast, an Austrian study involving 400 previously SARS-CoV-2-infected patients, of whom 72 developed PASC, found no EBV viremia in PASC patients and no significant differences in EBV serology between those with and without PASC [199]. Similar conclusions were reach by another study, which could not detect the presence of EBV using PCR in throat washings, stool, and blood samples from post-COVID-19 patients with neurological symptoms [83]. These studies collectively underscore the complex relationship between herpesvirus reactivation and the persistence of symptoms in post-COVID-19 conditions, highlighting the need for further research.

It is hypothesized that EBV reactivation can lead to a range of symptoms that overlap with or exacerbate those of PASC, including fatigue, neurocognitive dysfunction, and autonomic disturbances. EBV reactivation has been associated with neuroinflammation[116], which can contribute to the cognitive impairments observed in PASC patients. Microglial activation, a hallmark of neuroinflammation, can be driven by EBV reactivation and may lead to synaptic dysfunction, neuronal injury, and impaired neurogenesis [116]. These effects can manifest as brain fog, memory disturbances, and reduced cognitive function, which are commonly reported in PASC patients. EBV reactivation during acute SARS-CoV-2 infection can also contribute to PASC symptoms through promoting mitochondrial dysfunction [198].

Although herpesvirus reactivation has been proposed as a contributor to neurological symptoms, PCR results of blood or cerebrospinal fluid samples have not consistently confirmed this theory [75]. Additionally, Williams et al. conducted a study examining T cell responses against EBV and CMV in PASC patients with neurological symptoms compared with healthy individuals [195]. Their findings did not reveal significant differences in T cell responses between the cohorts [195]. However, they did observe an increased CD8 + T cell response to non-Spike antigens, although this difference has not been consistently confirmed in subsequent studies [195]. Specific tests for EBV or CMV did not establish a direct role of these viruses in the observed symptoms, leading Williams et al. to propose an indirect association mediated by systemic inflammation [195]. This suggests that while herpesvirus reactivation may not be the primary cause, it could contribute to the inflammatory milieu observed in PASC. Additionally, systemic effects of EBV reactivation include the promotion of a pro-inflammatory and pro-thrombotic state, which can exacerbate vascular and endothelial dysfunction. This can contribute to the persistent cardiovascular and systemic symptoms seen in PASC, such as fatigue and exercise intolerance. Understanding the role of EBV reactivation in the pathogenesis of PASC is crucial for developing comprehensive treatment strategies. Further research is needed to elucidate the mechanisms of EBV reactivation and its pathological consequences in the context of COVID-19 and PASC and to develop effective therapeutic interventions. This research should focus on the interplay between immune dysregulation, inflammatory responses, and viral reactivation to better understand and treat the complex symptomatology of PASC.

### Role of HHV-6 reactivation in the pathogenesis of PASC

After primary infection, usually in early childhood, HHV-6 establishes lifelong latency in various cell types, including monocytes, macrophages, and CD4 + T lymphocytes. Reactivation of HHV-6 under conditions of immune stress during or after an acute SARS-CoV-2 infection may significantly contribute to the pathogenesis of PASC [81, 82, 175, 177]. The ability of HHV-6 to infect endothelial cells and its neurotropic nature can have significant implications for PASC [148–150, 200]. Reactivation of HHV-6 in endothelial cells can potentially lead to endothelial dysfunction, contributing to microvascular impairments [200]. In the central nervous system, HHV-6 reactivation could potentially contribute to neuroinflammation and the neurocognitive symptoms observed in PASC. The role of HHV-6 as driver in the pathogenesis of ME/CFS is increasingly recognized and continuously re-emerging in scientific research [182, 189, 191, 192, 194, 201, 202]. The high prevalence of active HHV-6 infection in ME/CFS patients, along with the concurrent increase in plasma proinflammatory cytokines and the correlation between active viral infection and PASC-like clinical symptoms of ME/CFS, underscores the necessity for in-depth study of herpesvirus reactivation in the context of PASC to better understand its contribution to the pathogenesis of the disease [190, 193, 194, 203]. This growing body of evidence highlights the importance of exploring the role of HHV-6 and other herpesviruses in general and their microvascular impact in particular, in driving the complex symptomatology seen in PASC. Understanding these interactions is crucial for developing targeted interventions and improving patient outcomes [204].

## Conclusion and future direction for research

**Fig. 1:**
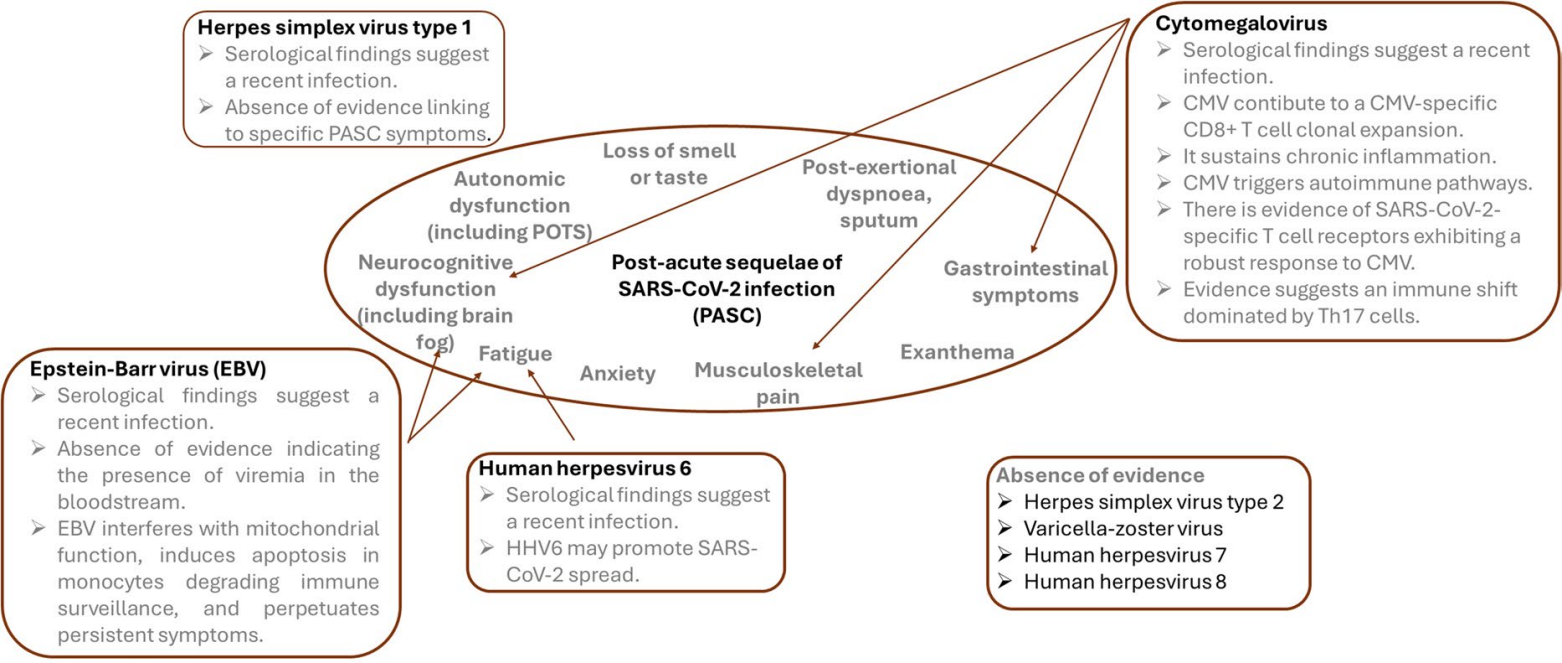
Summary of evidence concerning the associations between PASC and the herpesviridae family

PASC presents significant diagnostic challenges due to its diverse and often overlapping symptoms (Fig. 1).

The precise pathogenesis of post-acute sequelae of SARS-CoV-2 infection remains undetermined, complicating efforts to develop effective treatments. Numerous studies suggest that herpesvirus reactivation may contribute to PASC, although clinical trials have not consistently identified significantly elevated viremia levels. Nonetheless, evidence indicates that herpesviruses, particularly CMV, EBV, and/or HHV-6, may play an indirect role through mechanisms such as microvascular endothelial dysfunction, BBB disruption, immune dysregulation, and/or the maintenance of low-grade chronic inflammation. The direct infection of endothelial cells by SARS-CoV-2 and the potential reactivation of latent herpesviruses within these cells can contribute to the persistent vascular inflammation and dysfunction observed in PASC patients. This endothelial damage can impair neurovascular coupling, disrupt the BBB, and promote a pro-inflammatory state, exacerbating the symptoms of PASC. Additionally, elderly patients may be more affected by PASC due to age-related immunosenescence and impaired antiviral immune responses, which heighten their vulnerability to both SARS-CoV-2 and herpesvirus reactivation. Further research is essential to elucidate the exact mechanisms by which these viruses interact and contribute to the pathogenesis of PASC. Large-scale cohort studies and detailed mechanistic investigations are needed to confirm these findings and identify specific therapeutic targets. Developing targeted therapeutic strategies, such as antiviral treatments and anti-inflammatory agents, could mitigate the long-term effects of PASC and improve the quality of life for affected individuals.

## Data Availability

The data that support the findings of this study are available from the corresponding author, BGSz, upon reasonable request.
